# Hexokinase‐2 as a Therapeutic Target: Alleviating Herpes Simplex Keratitis Through Metabolic Reprogramming

**DOI:** 10.1002/advs.202503690

**Published:** 2025-06-20

**Authors:** Dan Jiang, Yining Sun, Xintong Yu, Ruoqi Wang, Yuting Zhang, Jinjie Yu, Shuang Xie, Yujia Cai, Yuhong Luo, Wei Chen

**Affiliations:** ^1^ National Clinical Research Center for Ocular Diseases Eye Hospital Wenzhou Medical University Wenzhou 325000 China; ^2^ State Key Laboratory of Ophthalmology Optometry and Visual Science Eye Hospital Wenzhou Medical University Wenzhou 325000 China; ^3^ Key Laboratory of Systems Biomedicine (Ministry of Education) Shanghai Center for Systems Biomedicine Shanghai Jiao Tong University Shanghai 200000 China; ^4^ State Key Laboratory of Medical Genomics Ruijin Hospital Affiliated to Shanghai Jiao Tong University School of Medicine Shanghai 200000 China; ^5^ Hangzhou Institute of Medicine (HIM) Chinese Academy of Sciences Hangzhou Zhejiang 310000 China

**Keywords:** herpes simplex keratitis, hexokinase‐2, lonidamine, metabolism, regeneration

## Abstract

Herpes simplex keratitis (HSK) is a leading infectious cause of blindness worldwide, with current therapies primarily targeting viral replication rather than addressing host‐cell injury. RNA sequencing of corneal tissue from HSK patients and healthy donors identifies a metabolic shift from mitochondrial oxidative phosphorylation to aerobic glycolysis. Notably, hexokinase‐2 (HK2), a pivotal glycolytic enzyme, exhibits the greatest up‐regulation, coinciding with a marked reduction in the activity of mitochondrial respiratory chain complexes in HSK corneas. Pharmacological inhibition of HK2 with lonidamine in human corneal epithelial cells reduces herpes simplex virus type 1 (HSV‐1) replication while preserving cell viability. In a murine model of HSK, topical lonidamine restored respiratory‐chain activity, lowered viral load, and accelerated corneal re‐epithelialization; its early therapeutic efficacy surpassed that of ganciclovir, and combination therapy conferred additive benefit. These findings identify HK2‐driven glycolytic reprogramming as a pathogenic hallmark of HSK and demonstrate that metabolic targeting concurrently restricts viral propagation and promotes tissue regeneration. Thus, metabolic intervention has the potential to complement direct antiviral therapy and represents a promising, clinically translatable strategy to preserve vision in HSK.

## Graphical abstract

Herpes Simplex Virus Type 1 (HSV‐1) infection triggers metabolic reprogramming in corneal cells, shifting from mitochondrial respiration to aerobic glycolysis with marked upregulation of Hexokinase‐2 (HK2). This study identifies HK2 as a therapeutic target: inhibiting HK2 with lonidamine significantly reduces viral replication and promotes corneal healing, highlighting host metabolic intervention as a promising strategy against herpes simplex keratitis.

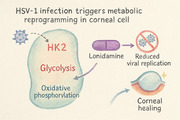

## 1 Introduction

Herpes simplex keratitis (HSK) is a recurrent inflammation of the cornea caused by herpes simplex virus type 1 (HSV‐1) infection and has emerged as a leading infectious cause of blindness globally.^[^ ^1^, ^2^ ^]^ Indeed, the infection rate of HSV‐1 is extensively documented, with the World Health Organization's data indicating that ≈67% of individuals under the age of 50 are affected on a global scale.^[^ ^1^, ^3^ ^]^ Population‐based studies from both Europe and North America place the annual incidence of HSV‐1 keratitis at roughly 10–15 cases per 100 000 inhabitants, with 13.2 / 100 000 reported in France and 11.8 / 100 000 in the United States.^[^ ^4^, ^5^ ^]^ Each year, an estimated 18–25 new cases occur per 100 000 people, and the recurrence rate is over 50% at 5 years and over 60% at 20 years.^[^ ^6^ ^]^ The disease is characterized by recurrent episodes of inflammation and tissue damage, leading to corneal scarring, vision loss, and potentially blindness.

Current treatment strategies for HSK primarily target viral replication with antiviral drugs such as acyclovir and ganciclovir. These drugs inhibit the viral DNA polymerase and help shorten the duration of HSK.^[^ ^7^ ^]^ Yet these agents have little effect on the immunopathogenic stromal phase, not eradicate latent HSV‐1, and therefore do not stop future relapses.^[^ ^8^ ^]^ Extended or high‐dose regimens select for acyclovir‐resistant strains, detected in up to 10% of ocular isolates and higher in immunocompromised patients.^[^ ^9^ ^]^ They also cause ocular surface toxicity and systemic complications like nephrotoxicity.^[^ ^9^, ^10^ ^]^ Crucially, conventional antivirals leave the virus‐induced host‐cell injury untouched; adjunctive corticosteroids are therefore required, yet corneal scarring and vision loss remain common.^[^ ^6^, ^7^, ^8^ ^]^ This underscores the critical need to develop new therapeutic strategies that target both the virus and the host‐cell responses in the pathogenesis of HSK.

HSV‐1 has a profound ability to hijack host‐cell gene expression, triggering a complex biological cascade that affects various cellular processes.^[^ ^11^, ^12^, ^13^ ^]^ The cornea, the primary site of infection, plays a crucial role in regulating these responses. Upon infection, the virus can induce apoptosis in corneal epithelial cells, leading to tissue damage and inflammation.^[^ ^8^, ^14^, ^15^ ^]^ These inflammatory responses contribute to the recruitment of immune cells and further exacerbate tissue damage.^[^ ^16^, ^17^ ^]^ Previous studies have suggested that HSV‐1 replication in corneal epithelial cells follows a distinct mechanism from that in other tissues, potentially involving the activation of apoptotic pathways to facilitate productive viral replication.^[^ ^18^, ^19^ ^]^ Additionally, recent studies demonstrate that HSV‐1 infection of the trigeminal ganglion in mice disrupts host metabolic homeostasis,^[^ ^20^ ^]^ including altered glutamate metabolism and polyamine pathway, both of which are critical for maintaining the latent and lytic viral cycles.^[^ ^21^, ^22^, ^23^ ^]^ Moreover, the limited understanding of the pathophysiology of HSV‐1‐induced ocular infections, due to reliance on animal models and a lack of focus on human ocular surface changes, further underscores the importance of studying HSV‐1 infection in human corneal tissues.^[^ ^24^, ^25^ ^]^ This is not only crucial for elucidating the molecular mechanisms of HSK but also for identifying potential therapeutic targets that can address both viral replication and host‐cell responses.

The objective of the present study is to investigate how host‐cell metabolism shapes HSK pathogenesis and to assess lonidamine‐mediated metabolic intervention as a therapeutic option. We first performed RNA sequencing on human corneal tissue from HSK patients and healthy donors to identify differentially expressed genes and pathways. Bioinformatic and functional screens converged on hexokinase‐2 (HK2)—a rate‐limiting glycolytic enzyme—as a central metabolic hub. We therefore evaluated the antiviral and tissue‐protective effects of pharmacologically inhibiting HK2 with lonidamine, a clinically tested HK2 inhibitor originally developed for oncology applications. Our data reveal a distinct glycolytic signature in HSK corneas and show that lonidamine‐driven HK2 blockade simultaneously reduces HSV‐1 replication and accelerates corneal healing. Together, these findings indicate that targeting host‐cell metabolism with lonidamine can complement direct antivirals and may lead to more effective, vision‐preserving treatments for HSK.

## 2 Results

### 2.1 Transcriptomic Profiling of Corneal Tissues from HSK Patients

**Figure 1:**
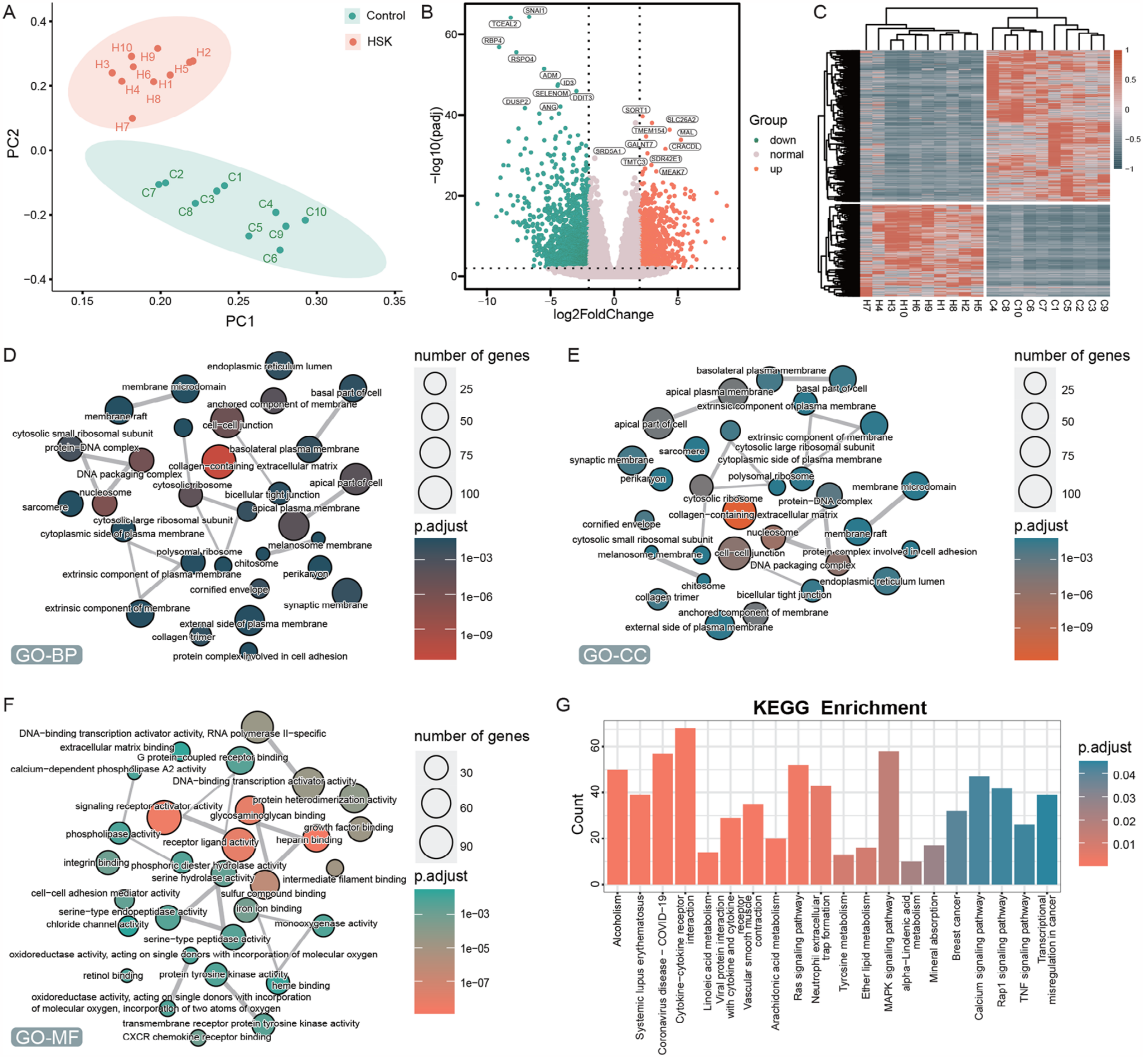
Differential gene analysis was performed between HSK and control group. A) PCA dimension reduction plot showing the different clusters between HSK patients and normal controls. B) Volcano graph showing upregulation and downregulation of genes. C) Heatmap showing upregulated and downregulated DEGs. D–F) GO and G) KEGG enrichment analysis of DEGs.

**Figure 2:**
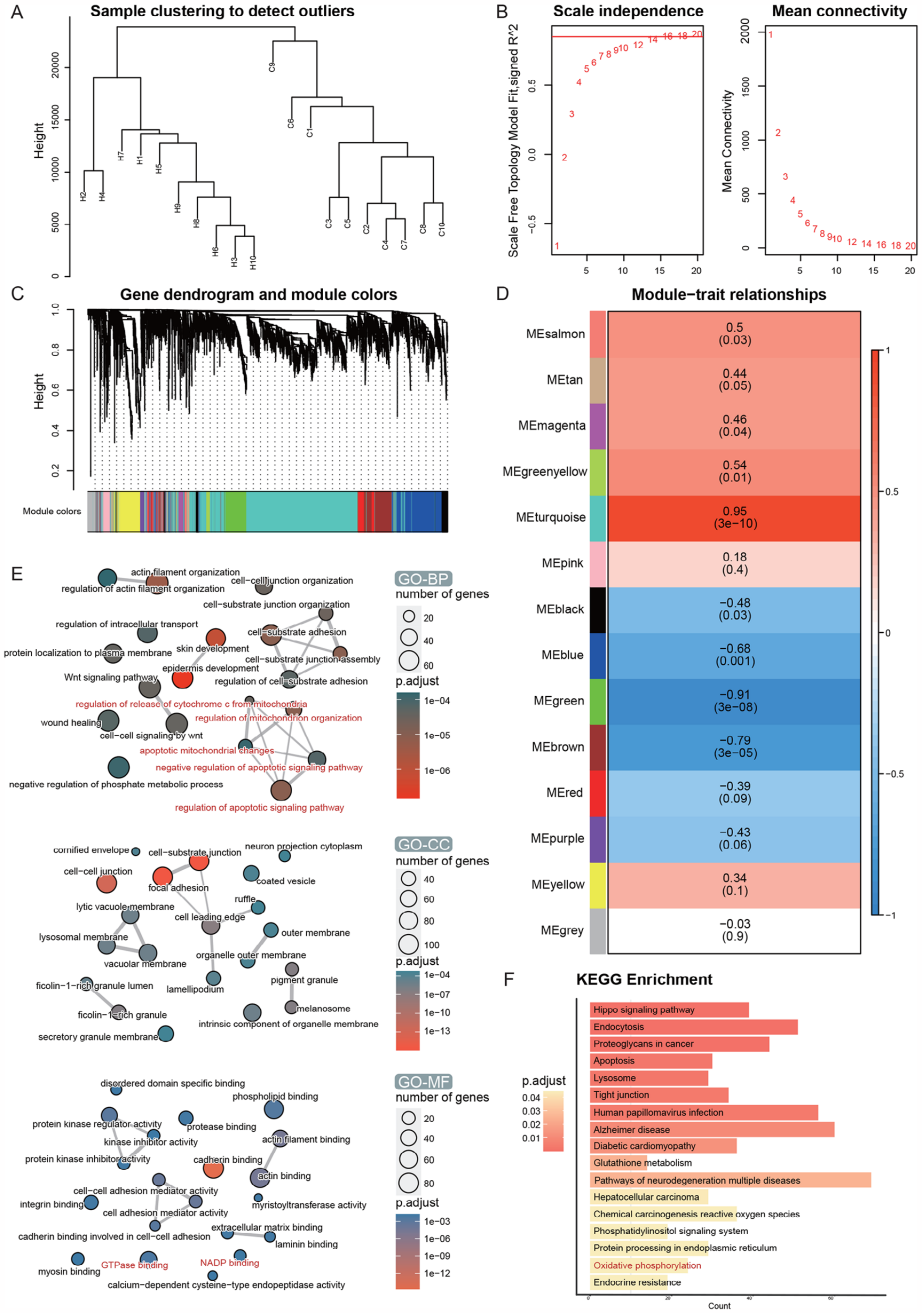
Gene expression dynamics and interaction networks in polyphenism modules. A) Sample clustering tree. B) Selection of the soft‐thresholding powers. C) Dendrogram of merged modules eigen genes obtained via WGCNA. The branches refer to clusters of genes that are highly connected. The colors in the horizontal bar represent the modules. D) This heatmap illustrates the correlation between modules (rows) and traits (columns), with positive correlations in red and negative correlations in blue. The left‐hand color swatches represent polyphenism modules. E) GO enrichment analysis of genes in the turquoise module. F) KEGG pathways enriched in the turquoise module.

To elucidate the transcriptomic alterations in HSK, we performed RNA sequencing on corneal samples from 10 HSK patients and 10 healthy donors. PCA clearly differentiated the two groups on the basis of mRNA profiles (Figure **1**A), revealing 2442 differentially expressed genes (DEGs; |log2(fold change)| ≥ 2, p_adjusted < 0.01), with 908 upregulated genes and 1534 downregulated genes (Figure 1B; Table S1, Supporting Information). Heatmap clustering revealed distinct mRNA and lncRNA expression patterns in HSK (Figure 1C). Analysis through the Gene Ontology (GO) and the Kyoto Encyclopedia of Genes and Genomes (KEGG) highlighted substantial alterations in the extracellular matrix composition, as well as diverse molecular pathways (Figure 1D–G). Weighted correlation network analysis (WGCNA) further segregated the HSK samples into 14 distinct gene coexpression modules, with the turquoise module showing the strongest correlation with HSK (Figure **2**A–D). To understand the biological roles of the turquoise module, we conducted enrichment analyses using GO and KEGG pathways. GO analysis suggested alterations in mitochondrial functions in HSK, with significant enrichment of processes related to cytochrome c release and apoptosis regulation (Figure 2E). The KEGG analysis confirmed these findings by highlighting enrichment in oxidative phosphorylation pathways (Figure 2F), indicating that HSK may involve disturbances in energy metabolism. These findings collectively suggest a complex transcriptomic response to HSV‐1 infection in corneal tissues, with significant alterations in cellular processes and pathways.

### 2.2 Identification of Key Metabolic Pathways in HSK

**Figure 3:**
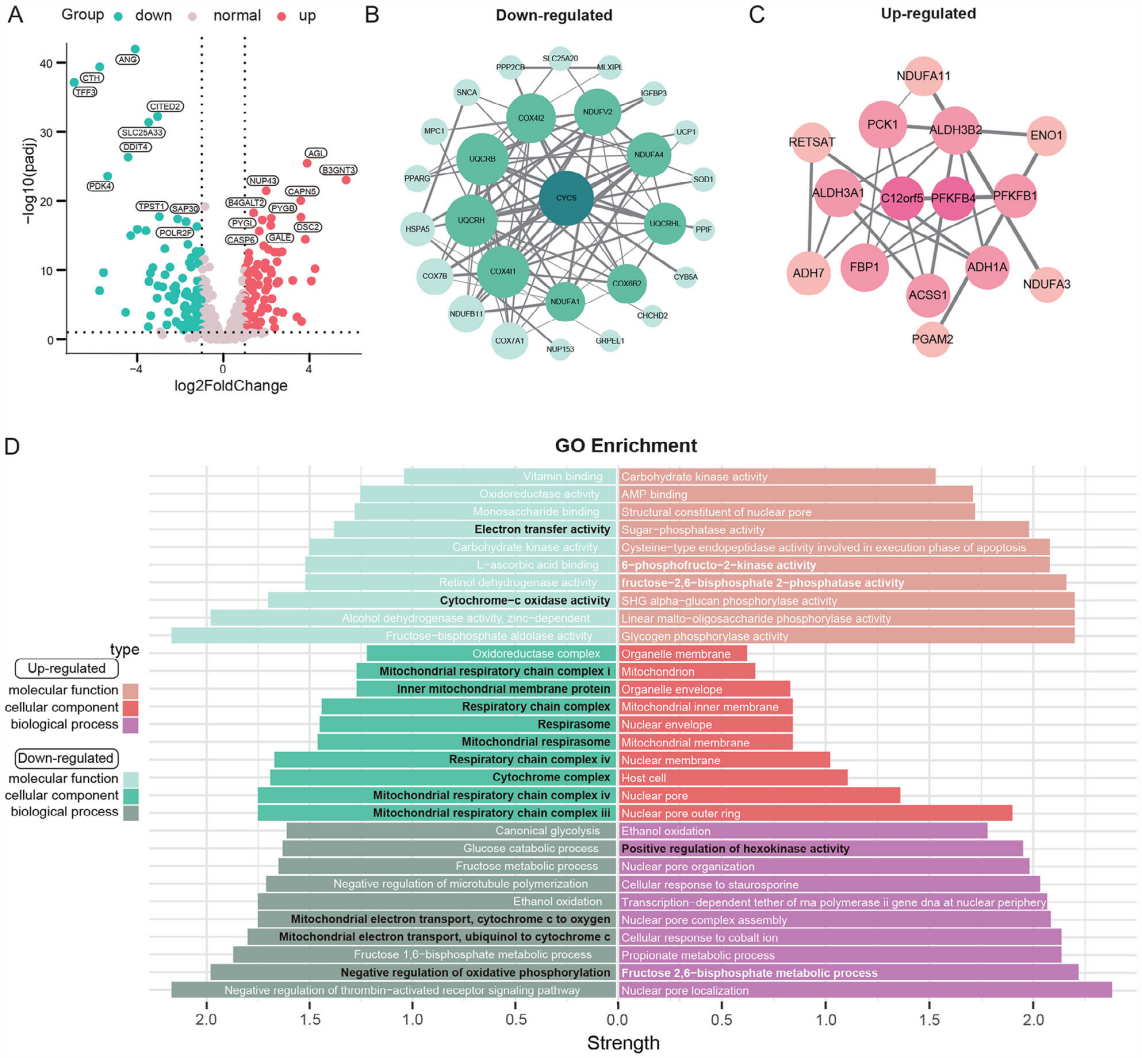
Changes of energy metabolism in HSK patients. A) Volcano plots of energy metabolism‐related genes in HSK, with blue indicating downregulated genes and red indicating upregulated genes. B) PPI network associated with the mitochondrial electron transport chain (ETC). C) PPI network linked to hexokinase enzymatic activity, mapping interactions between hexokinase and glucose metabolism. (Nodes represent ETC/hexokinase‐related proteins, with edge thickness reflecting interaction confidence scores.) D) Enrichment of GO pathways in PPI networks.

To evaluate alterations in metabolism, we examined the transcriptional profiles of genes associated with energy metabolic processes in HSK. Further analysis of energy metabolism revealed that when thresholds of |log2(fold change)| ≥ 1 and p_adjusted < 0.05 were used, a total of 173 genes were differentially expressed in the HSK group, with 82 genes upregulated and 91 genes downregulated (Figure **3**A; Table S2, Supporting Information). Utilizing the Search Tool for the Retrieval of Interacting Genes (STRING) repository, we constructed protein–protein interaction (PPI) networks for the genes associated with the significantly upregulated and downregulated DEGs, followed by topological analysis using the degree algorithm to identify and visualize hub nodes with high connectivity within these networks (Figure 3B,C). GO enrichment scrutiny of the proteins in the PPI networks revealed substantial suppression in pathways pertinent to mitochondrial oxidative phosphorylation. Notably, these encompassed the electron transport chain in mitochondria, specifically complexes I, III, and IV of the mitochondrial respiratory chain, as well as the broader respiratory chain complex (Figure 3D). Concurrently, there was an overexpression of HK, which is the rate‐limiting enzyme in the glycolysis pathway (Figure 3D). This finding, along with the downregulation of mitochondrial respiration pathways, suggests a potential metabolic reprogramming in corneal cells during HSK.

### 2.3 Role of HK2 in HSV‐1 Infection

**Figure 4:**
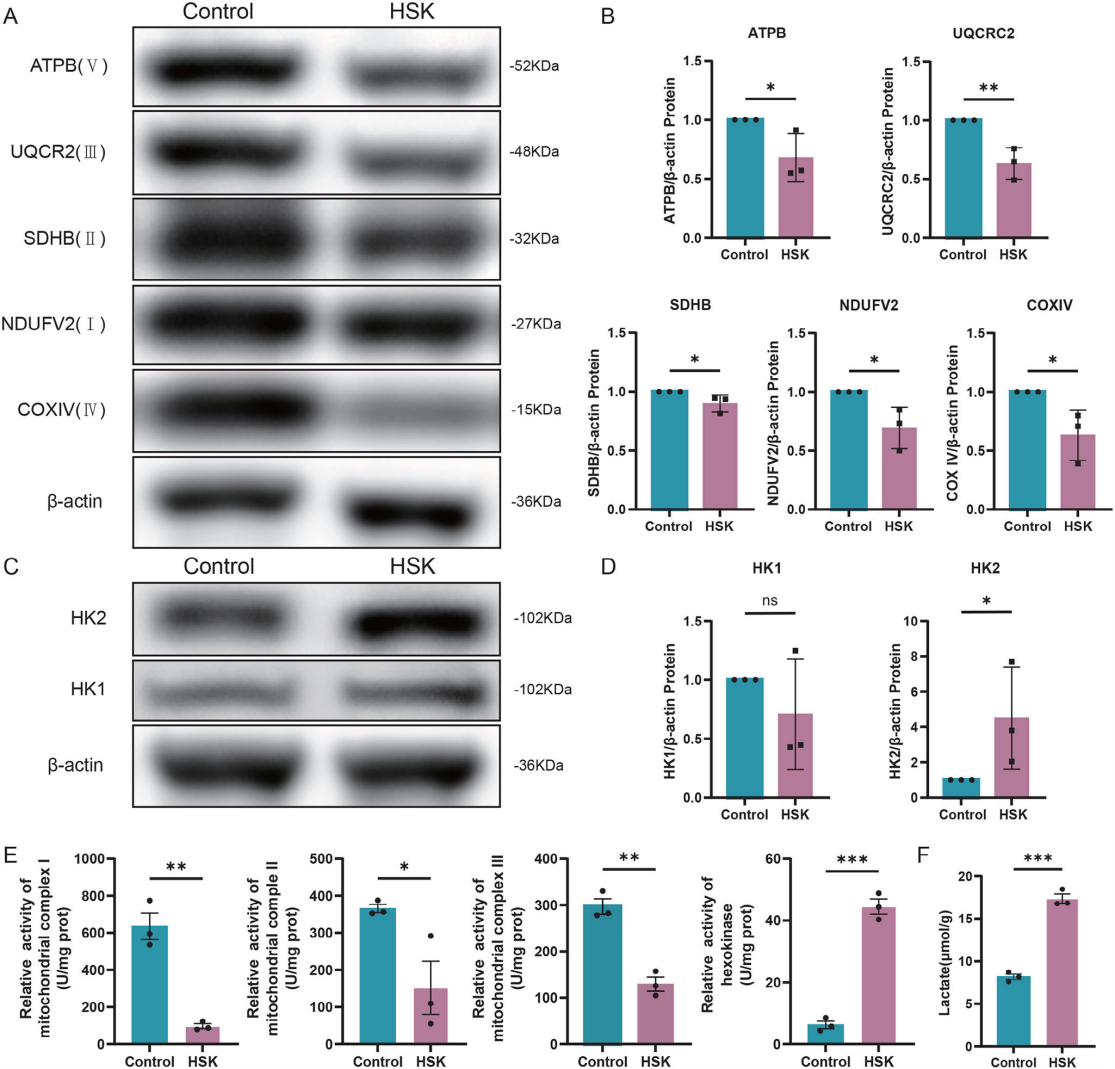
Expression analysis of respiratory chain complex proteins and HK in HSK patients and healthy controls. A,B) Western blot analysis and statistical quantification of respiratory chain complex‐related proteins, showing significantly reduced expression in HSK patients compared to normal controls (n=3). C,D) Western blot analysis and statistical quantification of HK1 and HK2 isoenzyme levels (n=3). E) Mitochondrial respiratory chain complex I, II, III, and HK activity (n=3). F) Lactic acid expression levels (n=3)(^*^ *P* < 0.05, ^**^ *P* < 0.01, ^***^ *P* < 0.001).

To confirm the hypothesis of metabolic reprogramming toward aerobic glycolysis in HSK, we examined the expression of mitochondrial respiratory chain complexes and HK in HSK and healthy corneas. Western blot analysis confirmed the downregulation of mitochondrial respiratory chain complex proteins (NDUFV2 (I), SDHB (II), UQCR2 (II), COXIV (IV), and ATPB (V)) and the upregulation of HK2 in HSK corneas (Figure **4**A–D). Functional assays revealed decreased activity of respiratory chain complexes I, II, and III, concurrent with elevated HK activity and increased extracellular lactate levels in HSK corneas (Figure 4E,F). These findings collectively support the notion of a shift from mitochondrial respiration to aerobic glycolysis in HSK corneal cells.

**Figure 5:**
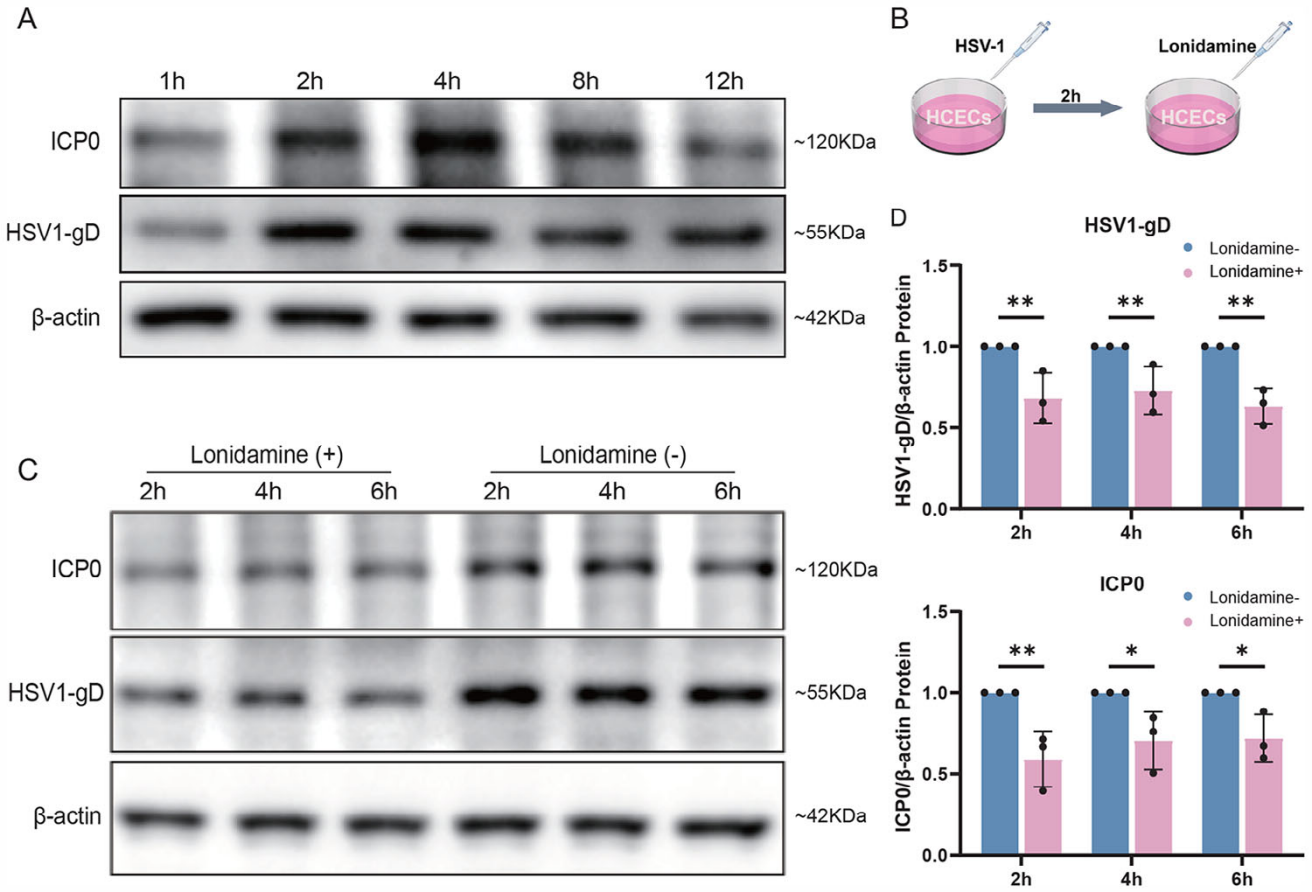
Evaluation of HSV1‐gD and ICP0 expression over time and following pharmacological intervention in vitro. A) Western blotting demonstrated that the protein expression of HSV1‐gD and ICP0 peaked at 2 to 4 hours post infection. B) Dosing 2 hours after infection. C) Representative western blotting image showing reduced protein expression of HSV1‐gD and ICP0 after lonidamine treatment. D) Statistical graphs showing the relative expression of the indicated proteins (n=3; ^*^ *P* < 0.05, ^**^ *P* < 0.01).

To investigate the role of HK2 in HSV‐1 infection and to explore potential therapeutic strategies targeting this enzyme, we utilized an in vitro model of corneal epithelial cells infected with HSV‐1(MOI = 1). We observed a peak in the expression of the viral replication markers HSV‐1 glycoprotein D (HSV1‐gD) and infected cell protein 0 (ICP0) in treated cells between 2 and 4 h post‐infection (Figure **5**A). This increase in viral protein expression was accompanied by a decline in cell viability, indicating the detrimental effects of HSV‐1 infection on the corneal epithelium (Figure 5A; Figure S1A, Supporting Information).

Considering the pivotal function of HK2 in cellular energy metabolism and its therapeutic potential as an antiviral target, we subsequently aimed to assess the influence of HK2 suppression on viral propagation. To this end, we first needed to determine the optimal concentration of lonidamine, a known HK2 inhibitor,^[^ ^26^ ^]^ for our treatments. Cell viability assays indicated that 100 µmol mL^−1^ is a safe concentration for lonidamine treatments (Figure S1B, Supporting Information). We treated the human corneal epithelial cells with lonidamine at 2 h post‐infection (Figure 5B), corresponding to the time point when viral replication is expected to be active. Subsequent to the treatment, we assessed the cells 2‐h later and detected significantly decreased expression of the viral replication markers HSV1‐gD and ICP0 in lonidamine‐treated cells compared to the untreated control cells (Figure 5C,D). These results suggest that targeting HK2 via lonidamine treatment can significantly inhibit viral replication in human corneal epithelial cells.

### 2.4 Therapeutic Efficacy of Lonidamine in HSK Mouse Model

**Figure 6:**
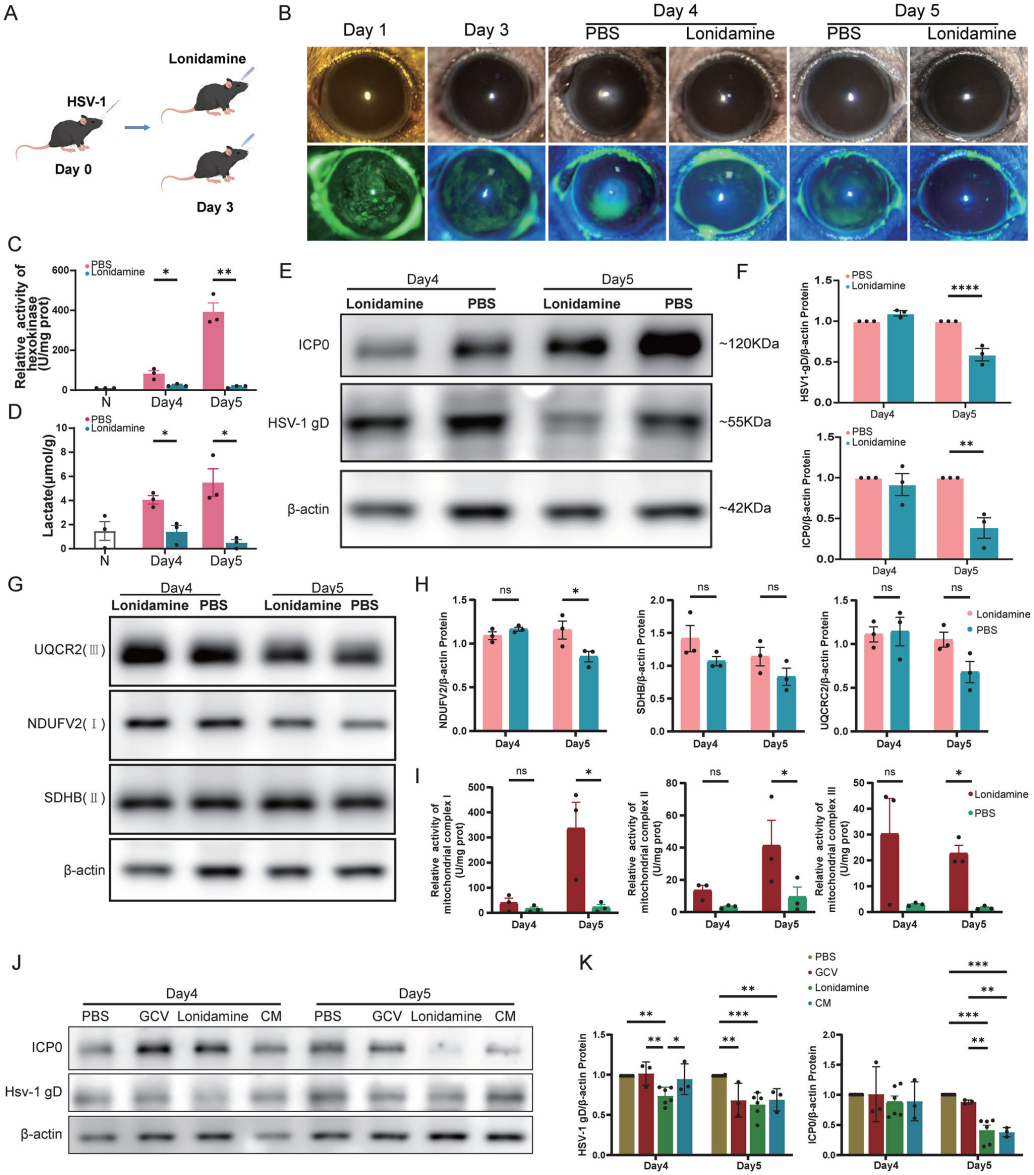
Effects of lonidamine on viral protein expression and metabolic enzymes in HSV‐1‐infected corneas in vivo. A) Dosing 3 days after infection. B) Photographs of corneas with and without fluorescein sodium staining after lonidamine treatment. C) HK activity after lonidamine treatment (n=3). D) Lactic acid expression levels. E) Representative WBs showing reduced protein expression of HSV1‐gD and ICP0 after lonidamine treatment. F) Statistical graphs showing HSV1‐gD and ICP0 protein expression (n=3). G) Representative western blotting image showing the protein expression of UQCR2, NDUFV2 and SDHB after lonidamine treatment. H) Statistical graphs showing the protein expression of UQCR2, NDUFV2 and SDHB (n=3). I) Enzyme activity of mitochondrial respiratory chain complexes I, II, and III (n=3). J) Representative western blotting image showing the protein expression of HSV1‐gD and ICP0 in different treatment groups (GCV: ganciclovir; CM: combination medication). K) Statistical graphs showing HSV1‐gD and ICP0 protein expression (PBS, Lonidamine: n=6; GCV, CM: n=3) (ns *P* > 0.05, ^*^ *P* < 0.05, ^**^ *P* < 0.01, ^**^ *P* < 0.001).

Having established the antiviral effects of HK2 inhibition in vitro, we extended our findings to an in vivo model to evaluate the therapeutic potential of targeting HK2 within the setting of HSV‐1‐induced keratitis. We assessed corneal damage and viral replication in the HSK mouse model, with peak severity observed on day 3 following HSV‐1 corneal infection (Figure S2A,B, Supporting Information). After establishing 500 µmol mL^−1^ as the optimal concentration of lonidamine through dose‐response studies (Figure S2C–E, Supporting Information), we topically applied the drug to mice cornea infected with HSV‐1 starting on day 3 post‐infection (Figure **6**A). This treatment resulted in significantly reduced corneal epithelial defects on days 4 and 5 post‐infection (Figure 6B). The lonidamine‐treatment group presented a significant decrease in HK enzyme activity (Figure 6C) and lactate production (Figure 6D). Additionally, the levels of viral replication markers HSV1‐gD and ICP0 in the corneas were notably decreased compared to controls (Figure 6E,F). Immunofluorescence staining further confirmed the suppression of HSV1‐gD expression specifically in the corneal epithelium after topical application of lonidamine (Figure S3, Supporting Information). Despite only slight changes in key proteins involved in the respiratory chain, such as NDUFV2, SDHB, UQCR2, and ATPB(Figure 6G,H), there was a significant increase in respiratory chain complex activity (Figure 6I). This finding indicates that HK inhibition by lonidamine successfully enhanced aerobic respiration, reversing the shift toward anaerobic glycolysis in HSK.

### 2.5 Comparison of Therapeutic Efficacy of Lonidamine and Ganciclovir Ophthalmic Gel

We next compared the therapeutic efficacy of lonidamine with that of the classic antiviral agent ganciclovir (GCV) ophthalmic gel for treating HSV‐1‐induced keratitis, evaluating both monotherapy and combination therapy (CM) approaches. The results indicated that lonidamine monotherapy allowed for earlier intervention, with a significant reduction in HSV1‐gD and ICP0 expression observed as early as 24 h post‐treatment (Day4) compared to the PBS control group (Figure 6J,K). Combination therapy, on the other hand, markedly decreases the suppression of ICP0 expression after 48 h (Day5), indicating a substantial decrease in viral replication. Both lonidamine monotherapy and the combination therapy showed superior efficacy to GCV monotherapy in the early stages of HSK in the mouse model (Figure 6J,K).

## 3 Discussion

Our findings demonstrate that herpes simplex keratitis (HSK) drives a pronounced metabolic reprogramming in the cornea, characterized by a shift from oxidative phosphorylation toward aerobic glycolysis. This virus‐induced metabolic switch is strikingly reminiscent of the Warburg effect classically observed in cancer cells,^[^ ^27^, ^28^, ^29^ ^]^ suggesting that HSV‐1 infection compels corneal cells to favor rapid glycolytic ATP production and biosynthesis at the expense of mitochondrial efficiency. Such an adaptation likely reflects the cellular response to the energetic and biosynthetic demands imposed by viral replication. Notably, we observed a robust upregulation of HK2 expression and activity in corneal tissues of HSK patients, implicating this rate‐limiting glycolytic enzyme as a key driver of the Warburg‐like phenotype in infected corneas. Our study provides fresh insight into HSK pathophysiology and highlights metabolic enzymes like HK2 as potential therapeutic targets to disrupt the disease process.

The metabolic shift observed in HSK appears to create a cellular environment highly favorable for viral proliferation. Viruses are well known to hijack host metabolism as a replication strategy,^[^ ^30^ ^]^ and HSV‐1 is no exception.^[^ ^31^ ^]^ Indeed, previous studies have shown that HSV‐1 infection can disrupt mitochondrial function‐for example, a viral nuclease (UL12.5) depletes mitochondrial DNA, leading to impaired electron transport and a marked reduction of ATP production.^[^ ^32^, ^33^ ^]^ Consistent with these reports, our HSK group exhibited significant down‐regulation of mitochondrial respiratory chain complexes, indicating that HSV‐1 infection causes substantial mitochondrial damage in the cornea. This virus‐induced mitochondrial dysfunction provides a direct mechanistic link to HSK pathogenesis: by crippling the host‐cell's major energy‐generating apparatus, HSV‐1 may drive the corneal tissue into an energy‐deficient, pro‐death state that contributes to ulceration and inflammation. In essence, what has been an underexplored aspect of keratitis, specifically the metabolic collapse of corneal cells, emerges as a pivotal event in disease progression, meriting further investigation.

Central to the observed glycolytic reprogramming is the role of HK2, which our data identify as markedly upregulated in HSV‐1‐infected human corneas. HK2 is a well‐recognized driver of enhanced glucose catabolism in rapidly proliferating cells,^[^ ^34^ ^]^ and many viruses similarly exploit this enzyme to meet their replication needs. In the context of HSV‐1, elevated HK2 expression would boost flux through glycolysis, supplying abundant ATP and metabolic intermediates to fuel viral DNA synthesis and virion assembly.^[^ ^35^ ^]^ In parallel, a high‐glycolytic state may undermine antiviral defenses: recent evidence indicates that accumulation of lactate (a byproduct of HK2‐driven glycolysis) can suppress innate immunity by post‐translationally modifying key immune sensors.^[^ ^36^ ^]^ Additionally, viral activation of HK2 has been shown to modulate immune signaling—for instance, one study found that an RNA virus co‐opts HK2 to trigger degradation of IRF3/7, thereby blunting interferon responses.^[^ ^37^, ^38^ ^]^ Taken together, these insights suggest that HK2 upregulation during HSK serves as a dual role: it provides metabolic fuel for HSV‐1 replication while concurrently helping the virus evade immune clearance. Accordingly, inhibiting HK2 is expected to hit the virus on multiple fronts, which is supported by our results showing that HK2 blockade led to lower lactate levels, restoration of a more oxidative metabolism, and a significant reduction in viral load in both cell culture and animal models of HSK.

An intriguing implication of our work is that HSK, despite being an inflammatory disease, exhibits metabolic hallmarks more commonly associated with malignancies. In this sense, HSK lesions metabolically mimic a “corneal cancer,” characterized by hyperactive glycolysis and suppressed mitochondrial respiration. We stress that this comparison is a conceptual one: unlike true cancers, the corneal cells in HSK are not transformed or autonomous, but their virus‐driven metabolic state does mirror the aggressive, glycolysis‐fueled phenotype of tumor cells. Recognizing this parallel is valuable, as it means we can potentially repurpose strategies from cancer metabolism research to manage HSK. By targeting HK2 via lonidamine, our therapeutic strategy aims to cut off the virus's “fuel supply,” thereby directly limiting HSV replication and also indirectly reducing the excessive inflammatory response. By illuminating this previously unappreciated aspect of HSK pathogenesis, our study broadens the framework for developing treatments, emphasizing that effective management of HSV‐1 ocular infections may require not just antiviral activity, but also correction of the host's metabolic imbalance.

Looking ahead, translating these insights into clinical practice requires further validation. First, the mouse model only reflects acute infection and inflammation, while human HSV‐1 can establish latency and reactivate episodically. Second, although our ex vivo and in vivo results are promising, future studies need to include larger and more diverse HSK patient cohorts to confirm the efficacy of HK2‐focused interventions across different genetic backgrounds and disease stages. Long‐term safety is also critical, as metabolic inhibitors may affect healthy cells. Finally, in‐depth mechanistic studies are needed to understand how HK2 inhibition interacts with host immune responses and corneal wound healing over time. These steps are essential for establishing metabolism‐centered therapy as a viable and safe addition to the current HSK treatment options and moving toward clinical trials to improve patient outcomes.

## 4 Experimental Section

### Patients and Clinical Evaluation

This study received ethical approval from the Human Ethics Committee at Eye Hospital of Wenzhou Medical University (YSG202250). The trial was registered in the Chinese Clinical Trial Registry (ChiCTR2200060105) and conducted in strict accordance with the principles outlined in the Declaration of Helsinki and Good Clinical Practice (GCP) guidelines. Study participants were recruited from the Eye Hospital of Wenzhou Medical University. The collected clinical data included demographic information (age, sex), age at HSK diagnosis, and any previous ocular conditions. HSK status was independently evaluated by two ophthalmologists via slit lamp biomicroscopy. The assessments documented the visual acuity using a standard LogMAR chart, as well as the corneal vascularization and ocular surface conditions through detailed photographic records. Patients were rigorously selected based on established inclusion and exclusion criteria, with those having a history of conditions such as ocular surgery, uveitis, glaucoma, or ocular trauma being excluded from the study.

### Sample Collection and Processing

Corneal tissues were excised from patients with HSK during keratoplasty procedures to form the experimental group. Control samples were harvested from subjects who underwent corneal refractive surgery for myopia at the Eye Hospital of Wenzhou Medical University. Following the surgical operation, corneal tissues were quickly transferred to precooled cryopreservation tubes and stored at −80 °C until the initiation of RNA extraction procedures.

### RNA Purification and Library Construction

To elucidate the transcriptomic landscape, RNA samples from 10 HSK‐affected individuals and 10 healthy donors were subjected to RNA sequencing library preparation. Total RNA was extracted from frozen tissues via TRIzol reagent (Invitrogen Life Technologies, USA), adhering to the standard acid‐guanidinium‐phenol‐chloroform methodology. RNA quality control involved multiple steps: 1% agarose gel electrophoresis for degradation and contamination assessment, NanoPhotometer spectrophotometry (IMPLEN, CA, USA) for purity evaluation, and the RNA Nano 6000 Assay Kit on an Agilent Bioanalyzer 2100 system (Agilent Technologies, CA, USA) for integrity analysis. RNA sequencing libraries were prepared from 1 µg of total RNA per sample using the NEBNext Ultra RNA Library Prep Kit for Illumina, following the manufacturer's standardized procedures. Post‐amplification purification was conducted using the AMPure XP system, followed by library quality assessment on an Agilent Bioanalyzer 2100. Libraries were processed for cluster generation with the TruSeq PE Cluster Kit v3‐cBot‐HS (Illumina) under standardized conditions, then sequenced on an Illumina NovaSeq platform.

Raw sequencing outputs underwent base calling through CASAVA to generate FASTQ‐formatted reads. Preprocessing removed adapter contaminants and low‐quality sequences (Q20 ≥ 98.09%) prior to downstream analysis. Alignment against Ensembl reference genomes (http://www.ensembl.org) was performed using HISAT2 v2.0.5, with gene‐level quantification via FeatureCounts 1.5.0‐p3. The dataset comprised 43,388,492 reads/sample (average), demonstrating high mapping efficiency (98‐99% to human genome GRCh38). A total of 58337 expressed mRNAs and 12228 lncRNAs were detected from the 20 samples.

Differential gene expression between the two groups was analyzed using the DESeq2 tool (version 4.1.0). Significant DEGs were identified with thresholds of p_adjusted < 0.01 and |log2(fold change)| ≥ 2. GO and KEGG enrichment analyses were conducted via the R package cluster Profiler (https://guangchuangyu.github.io/clusterProfiler), which incorporates gene length bias correction. Functional enrichment significance was defined as Gene Ontology (GO) terms and KEGG pathways exhibiting adjusted *p*‐values < 0.05.

Weighted gene co‐expression network analysis 76 was performed in R using the WGCNA package. The analysis was performed on normalized gene expression data (log2 counts per million), focusing on the 5000 most variable RNAs extracted from the HSK samples. The hybrid cut‐tree algorithm was employed for module generation, with no merging performed. Module eigengenes were calculated, and their correlation with HSK was assessed via biweight midcorrelation analysis. The module with the strongest correlation was selected for further study. The functional significance of the selected module was assessed using GO and KEGG enrichment analyses conducted with the R package cluster Profiler on all modules.

To identify gene sets associated with energy metabolism, the Molecular Signatures Database (MSigDB, http://software.broadinstitute.org/gsea/msigdb/) was utilized. The analysis spanned 29 gene sets (Table S3, Supporting Information), yielding 592 overlapping genes for differential expression analysis. Protein‒protein interaction (PPI) networks were constructed for both upregulated and downregulated DEGs via the STRING database (http://string‐db.org). All proteins within the PPI networks were subjected to GO enrichment analysis.

### Cells and Virus

Human corneal epithelial cells (HCECs, the 12‐SV40 immortalized HCE‐2 line) were purchased from the American Type Culture Collection (Manassas, VA, USA). Cell culture was conducted via Dulbecco's Modified Eagle Medium/Nutrient Mixture F‐12 (DMEM/F‐12) enriched with 10% fetal bovine serum (FBS), insulin–transferrin–selenium (ITS, 5 µg mL^−1^, and 1% penicillin–streptomycin. The HSV‐1 (McKrae strain) was sourced from Shanghai Jiao Tong University School of Medicine, China. Viral amplification and titration were performed using Vero cells. The medium used for Vero cells was DMEM (HyClone, Logan, Utah, USA) supplemented with 10% FBS (PAN Biotech, Edenbach, Bagoria, Germany).

### Cell Viability Assay

To assess the dose‐dependent effects on cell viability, HCECs were inoculated into 96‐well plates at 1.0 × 10^4^ cells per well and incubated for 24 h. The cells were subsequently exposed to a gradient of different concentrations of lonidamine for 12 h. After washing twice with PBS and incubating for 2 h in 10% Cell Counting Kit‐8 (CCK‐8), the absorbance value at 450 nm was measured with an enzyme standard (Molecular Devices, CA, USA).

### HSV‐1 Ocular Infection in Mice

This study was approved by the Laboratory Animal Ethics Committee at Wenzhou Medical University and the Laboratory Animal Centre at Wenzhou Medical University (YSG24092903). Six‐ to eight‐week‐old C57BL/6 mice were purchased from Jiesijie Laboratory Animal Company (Shanghai, China). Mice were included in the study on the basis of the following criteria: absence of corneal opacities, no evidence of ocular infections or ulcerations upon slit‐lamp examination, and corneal fluorescein staining scores < 8. Corneal infection procedures were performed on tribromoethanol‐anesthetized mice (12.5% solution, 0.25 mL/10 g body weight via intraperitoneal injection). The corneas of the mice were lightly scarified with a needle (26 G × 3/8 in.) 10 times, and a 4‐µL drop containing 2 × 10^8^ plaque‐forming units (PFUs) of HSV‐1 was applied to the eye.

### Drug Sources and Specifications

Lonidamine was obtained from MedChemExpress (HY‐B0486) and GCV ophthalmic gel (0.15%) from Hubei Keyi Pharmaceutical Co. All treatments were administered three times daily (8:00 AM, 2:00 PM, and 8:00 PM) for 2 consecutive days, with 4 µL of lonidamine solution and 2 µL of GCV ophthalmic gel applied per eye. For combination therapy, a 10‐min interval was maintained between applications, with lonidamine eye drops applied first.

### Western Blot

Cells and corneal tissues were lysed using radiation immunoprecipitation assay buffer (Solarbio, Beijing, China). Protein concentrations were quantified with a BCA assay kit (Beyotime, Beijing, China), followed by SDS‐PAGE separation of equal protein quantities (GE Healthcare Life Sciences, USA). Membranes were blocked with 5% milk (2 h), then incubated overnight at 4 °C with primary antibodies. After 2 h room‐temperature exposure to HRP‐conjugated secondary antibodies, chemiluminescent detection was performed using Beyotime reagents. Immunoblots were digitized on a GE Amersham Imager AI680. The primary antibodies used were as follows: HSV1‐gD (Abcam, ab6507), ICP0 (Abcam, ab6513), HK1(Proteintech, 19662‐1‐AP), HK2 (Proteintech, 66974‐1‐Ig), NDUFV2 (Proteintech, 15301‐1‐AP), UQCRC (Proteintech, 214742‐1‐AP), ATPB (Abcam, ab14730), COX IV (Abcam, ab33985), and SDHB (Proteintech, 10620‐1‐AP).

### Hematoxylin and Eosin (H&E) Staining

Corneal specimens were cryopreserved in optimal cutting temperature (OCT) compound prior to sectioning. Sequential 10‐µm frozen sections underwent fixation with 4% paraformaldehyde (10 min) followed by two distilled water rinses (2 min each). Staining protocol comprised hematoxylin immersion (5–10 min) with subsequent tap water differentiation (10 min), brief distilled water wash, and eosin counterstaining (1 min). Dehydration was achieved through a graded ethanol series (70%, 80%, 90%, 100%; 10 s each) prior to xylene clearing (2 × 5 min). Histomorphological evaluation was conducted using brightfield microscopy.

### Measurement of Enzyme Activity

HK activity and mitochondrial electron transport chain complexes I–III were quantified using commercial assay kits (HK Activity Assay Kit; Mitochondrial Complex I/II/III Activity Assay Kit, Solarbio). Corneal tissues (equal mass) were homogenized in ice‐cold extraction buffer, followed by sequential centrifugation (600 ×g, 10 min, 4 °C for debris removal; 11 000 ×g, 15 min, 4 °C for supernatant isolation). Reaction mixtures containing sample lysates and kit reagents were analyzed spectrophotometrically at 450 nm using a Thermo Fisher microplate reader. Enzyme activities were derived from standard curves according to the manufacturer's protocols.

### Lactate Production

The lactate content in the corneal tissues was measured with an LA Content Assay Kit (Solarbio, BC2235). Lactate extraction from equivalent sample quantities across different groups was performed via extraction solutions A and B. According to manufacturer specifications, extracted analytes were mixed with chromogenic reagent in 96‐well plates. The absorbance was measured at 570 nm to determine the lactate concentration.

### Immunofluorescence Staining

Corneal immunofluorescence was performed on euthanized mice following enucleation. Eyeballs were OCT‐embedded, cryosectioned at 10 µm thickness, and fixed in 4% paraformaldehyde (15 min, RT). After PBS washes (3×), tissue permeabilization used 0.4% Triton X‐100/PBS (20 min), with subsequent blocking in 10% normal goat serum (2 h). The tissue sections were incubated with primary antibodies overnight at 4 °C, followed by fluorescence labeling with secondary antibodies and immunoimaging via a confocal microscope. The following antibodies were used for immunofluorescence: HSV1‐gD (Abcam, ab6507) and HK2 (Proteintech, 66974‐1‐Ig).

### Statistical Analysis

Experimental data represent triplicate biological replicates (mean ± SD). Statistical significance was determined by Student's *t*‐test in GraphPad Prism 10, with thresholds defined as ^*^ *P* ≤ 0.05, ^**^ *P* ≤ 0.01, ^***^ *P* < 0.001, and ^****^ *P* ≤ 0.0001.

## Conflict of Interest

The authors have a patent application, CN202410259015.3, titled ‘Application of inhibiting hexokinase isoenzyme 2 in the treatment of herpes simplex keratitis’ with W.C., D.J., and Y.N.S. as co‐inventors, filed by Affiliated Eye Hospital of Wenzhou Medical University; Y.J.C. is a co‐founder and advisor of BDGENE Therapeutics.

## Author Contributions

D.J. and Y.S. contributed equally to this work. D.J. and W.C. designed the research; Y.S. and D.J. analyzed the data; Y.S., X.T.Y., R.Q.W., Y.T.Z., J.J.Y., and S.X. performed the experiments and collected the data; Y.J.C. provided the HSV‐1 virus and consultation on experimental operations; D.J. and Y.S. drafted the manuscript; W.C., D.J. and Y.L. conceived and supervised the study and revised the manuscript. All authors read and approved the final manuscript.

## Supporting information



Supporting Information

Supplemental Table 1

Supplemental Table 2

Supplemental Table 3

## Data Availability

The data that support the findings of this study are openly available in China National Center for Bioinformation at https://ngdc.cncb.ac.cn/gsa‐human/browse/HRA009034, reference number 9034.
