# Supplementary material for: Hexokinase‐2 as a Therapeutic Target: Alleviating Herpes Simplex Keratitis Through Metabolic Reprogramming
Source: Adv Sci (Weinh). 2025 Jun 20;12(34):e03690. doi: 10.1002/advs.202503690 (PMC12442686; doi:10.1002/advs.202503690)
Supplement: Supplementary file 1 — Supporting Information [file ADVS-12-e03690-s001.docx]

**
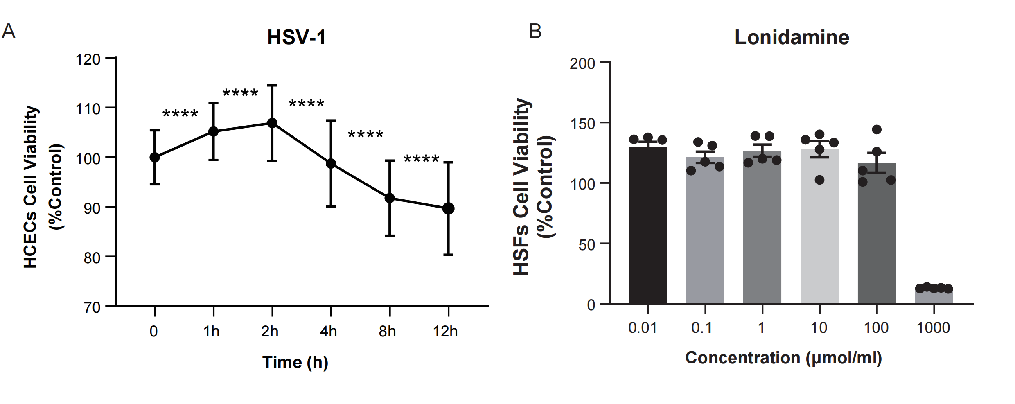
Supplementary Figures**

**Supplementary Figure 1.** (A) Quantification of human corneal epithelial cell (HCEC) viability at different times post infection (n=3; ****P < 0.0001). (B) Quantification of HCEC viability after treatment with different concentrations of lonidamine (n=5).


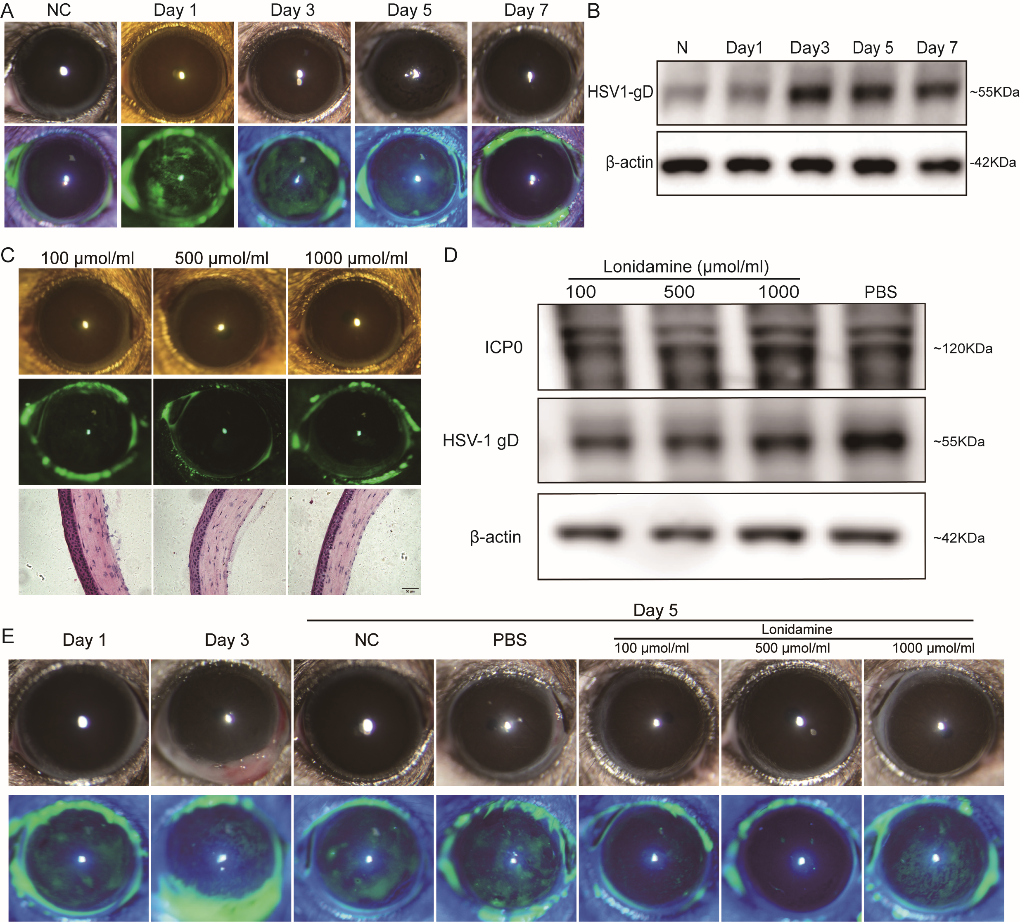


**Supplementary Figure 2.** (A) Photographs of corneal staining and nonstaining at different times after HSV-1 infection. (B) Representative western blot showing the protein expression of HSV1-gD at different times after HSV-1 infection. (C) Photographs of corneas with and without fluorescein sodium staining and HE staining images of mice treated with different concentrations of lonidamine. (D) Representative Western blot showing the protein expression of HSV1-gD and ICP0 after treatment with different concentrations of lonidamine. (E) Images of corneal staining and nonstaining with different concentrations of lonidamine after 48h of treatment.


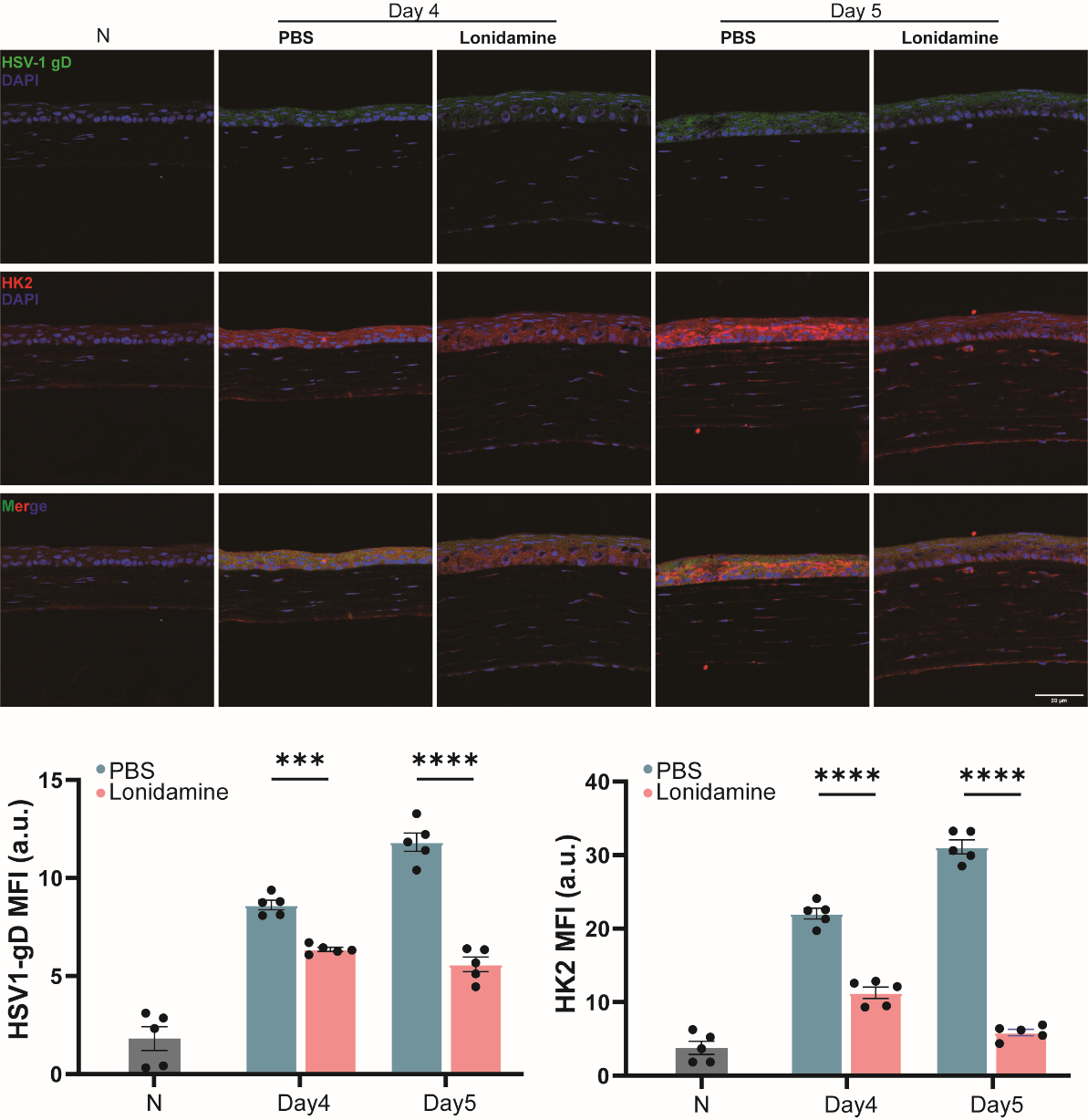


**Supplementary Figure 3.** Immunofluorescence staining revealed that lonidamine treatment reduced HK2 and HSV1-gD expression in the mouse corneal epithelium after HSV-1 infection. Statistical graphs showing the expression of these genes (n=5; ***P < 0.001,****P < 0.0001).

**Supplementary Table 1.** Differentially expressed genes

**Supplementary Table 2.** Differential expression of metabolic genes

**Supplementary Table 3.** Energy metabolism related gene sets
